# RNA-seq dataset of subcutaneous adipose tissue: Transcriptional differences between obesity and healthy women

**DOI:** 10.1016/j.dib.2021.107647

**Published:** 2021-11-27

**Authors:** Letizia Messa, Federica Rey, Cecilia Pandini, Bianca Barzaghini, Giancarlo Micheletto, Manuela Teresa Raimondi, Simona Bertoli, Cristina Cereda, Gianvincenzo Zuccotti, Raffaella Cancello, Stephana Carelli

**Affiliations:** a Department of Chemistry, Materials and Chemical Engineering ``Giulio Natta'', Politecnico di Milano, Milano, Italy; b Department of Biomedical and Clinical Sciences ``L. Sacco'', University of Milan, Via Grassi 74, 20157 Milan, Italy; c Pediatric Clinical Research Centre Fondazione “Romeo ed Enrica Invernizzi”, University of Milano, Milano, Italy; d Genomic and post-Genomic Centre, IRCCS Mondino Foundation, 27100 Pavia, Italy; e Department of Pathophysiology and Transplantation, INCO and Department of General Surgery, Istituto Clinico Sant'Ambrogio, University of Milan, Milan, Italy; f Obesity Unit—Laboratory of Nutrition and Obesity Research, Department of Endocrine and Metabolic Diseases, IRCCS Istituto Auxologico Italiano, Milan, Italy; g International Center for the Assessment of Nutritional Status (ICANS), Department of Food, Environmental and Nutritional Sciences (DeFENS), University of Milan, Milan, Italy; h Department of Women, Mothers and Neonatal Care, Children's Hospital ``V. Buzzi'', Milan, Italy; i Department of Pediatrics, Children's Hospital ``V. Buzzi'', Milan, Italy

**Keywords:** RNA-Seq analysis, Transcriptome analysis, Deregulated pathways, GSEA, R Studio, UMI

## Abstract

In this data article, we present the dataset from the RNA-Seq analysis of subcutaneous adipose tissue collected from 5 healthy normal weight women (NW, age 37 ± 6.7 years, BMI 24.3 ± 0.9 kg/m^2^) and 5 obese women (OBF, age 41 ± 12.5 years, BMI 38.2 ± 4.6 kg/m^2^). Raw data obtained from Illumina NextSeq 500 sequencer were processed through BlueBee® Genomics Platform while differential expression analysis was performed with the DESeq2 R package and deposited in the GEO public repository with GSE166047 as accession number. Specifically, 20 samples divided between NW (control), OBF (obese women), OBM (obese male) and OBT2D (obese women with diabetes) are deposited in the GSE166047. We hereby describe only 10 samples (5 healthy normal weight women reported as NW and 5 obese women reported as OBF) because we refer to the data published in the article “Transcriptional characterization of Subcutaneous Adipose Tissue in obesity affected women highlights metabolic dysfunction and implications for lncRNAs” (DOI: 10.1016/j.ygeno.2021.09.014). Pathways analyses were performed on g:Profiler, Enrichr, ClueGO and GSEA to gain biological insights on gene expression. Raw data reported in GEO database along with detailed methods description reported in this data article could be reused for comparisons with other datasets on the topic to obtain transcriptional differences in a wider co-hort. Moreover, detailed pathways analysis along with cross-referenced data with other datasets will allow to identify novel dysregulated pathways and genes responsible for this regulation. The biological interpretation of this dataset, along with related in vitro experiments, is reported by Rey et al., in Genomics (DOI: 10.1016/j.ygeno.2021.09.014).

## Specifications Table

**Table:** 

Subject	Biological and Molecular Sciences
Specific subject area	Transcriptomics Category: description of RNA-sequencing data obtained from whole transcriptome analysis.
Type of data	Table Image Fig.
How data were acquired	RNA-Sequencing and differential expression analysis
Data format	Raw data deposited in GEO database Analysed Filtered
Parameters for data collection	Biopsies of subcutaneous adipose tissue collected from 5 healthy normal weight women (NW, age 37 ± 6.7 years, BMI 24.3 ± 0.9 kg/m^2^) and 5 obese women (OBF, age 41 ± 12.5 years, BMI 38.2 ± 4.6 kg/m^2^)
Description of data collection	RNA-Seq analysis of subcutaneous adipose tissue of obese and healthy women
Data source location	Institution: University of Milan City: Milan Country: Italy Latitude and longitude for collected samples/data: 45.5189° N, 9.1227° E
Data accessibility	Repository name: Gene Expression Omnibus Data identification number: GSE166047 Direct URL to data: https://www.ncbi.nlm.nih.gov/geo/query/acc.cgi?acc=GSE166047 We hereby describe only 10 samples (5 healthy normal weight women reported as NW and 5 obese women reported as OBF) of the 20 samples because we refer to the published article that follows
Related research article	F. Rey, L. Messa, C. Pandini, B. Barzaghini, G. Micheletto, M.T. Raimondi, S. Bertoli, C. Cereda, G.V. Zuccotti, R. Cancello, S. Carelli, **Transcriptional characterization of Subcutaneous Adipose Tissue in obesity affected women highlights metabolic dysfunction and implications for lncRNAs** Genomics. 2021 Sep 21;113(6):3919-3934. doi: https://doi.org/10.1016/j.ygeno.2021.09.014. Epub ahead of print. PMID: 34555498.

## Value of the Data

• The data reported here provide the full transcriptional profile of subcutaneous adipose tissue from obesity affected women compared to healthy controls. Raw data analysis could highlight novel targets and regulators in obesity. Moreover, the analysed data provides insights into the roles of genes differentially expressed in this dataset, with an in-silico dissection of their predicted functions and implications.
• The detailed pathway enrichment analysis performed both with Enrichr, g:Profiler, Cytoscape and GSEA provides a comprehensive characterization of the potential pathways and gene ontologies in which the DE RNAs are involved. This will help researchers
• identify novel dysregulated pathways in obesity. Moreover, it will help identify the genes which are causative for this dysregulation.
• Molecular biologists will have new data for the characterization of adipogenic dysfunctions in obesity-affected patients, with specific directions on the processes in which these genes are implicated. Moreover, the present work could provide new biomarkers and targets of disease progression to be looked out for clinical practice.
• Other researchers and clinicians could benefit from these data for wider cohort analysis. The raw data reported here could be re-processed and cross-referenced with other datasets on the topic to provide a comprehensive characterization of the differential expression in subcutaneous adipose tissue in obesity. Moreover, the data could be of reference for future in vitro validation, directing scientists towards the possible biological functions of the genes.

## 1 Data Description

Subcutaneous adipose tissue (SAT) from five healthy women (NW_1, NW_2, NW_3, NW_4, NW_5) and five obese women (OBF_1, OBF_2, OBF_3, OBF_4, OBF_5) was subjected to RNA-Seq and the present dataset reports the analysis. Raw data obtained from Illumina NextSeq 500 sequencer were deposited as FASTQ and gene abundance data in Gene Expression Omnibus (GEO) database (accession number: GSE166047). Specifically, 20 samples divided between NW (Control), OBF (obese women), OBM (obese male) and OBT2D (obese women with diabetes) are deposited in the GSE166047. The present dataset describes only 10 samples (5 healthy normal weight women reported as NW and 5 obese women reported as OBF) of the 20 samples because we refer to the data published in the article “Transcriptional characterization of Subcutaneous Adipose Tissue in obesity affected women highlights metabolic dysfunction and implications for lncRNAs” (DOI: 10.1016/j.ygeno.2021.09.014). The accession number for individual samples in GEO database were presented as GEO run accession number in Table 1. By mapping each sample to human reference genome, the number of mapped reads was estimated (Table 2).

**Table 1:** List of accession number of obesity and healthy women transcriptome in GEO database.

Sample	Phenotype	GEO accession number	GEO run accession number
NW_1	Healthy	GSM5060703	SRR13615650
NW_2	Healthy	GSM5060704	SRR13615651
NW_3	Healthy	GSM5060705	SRR13615652
NW_4	Healthy	GSM5060706	SRR13615653
NW_5	Healthy	GSM5060707	SRR13615654
OBF_1	Obese	GSM5060708	SRR13615655
OBF_2	Obese	GSM5060709	SRR13615656
OBF_3	Obese	GSM5060710	SRR13615657
OBF_4	Obese	GSM5060711	SRR13615658
OBF_5	Obese	GSM5060712	SRR13615659

**Table 2:** Summary statistics of reads mapping and transcripts assembly for each obesity and healthy women sample.

Sample	Uniquely mapped	Mapped to multiple loci	Mapped to too many loci	Unmapped: too short	Unmapped: other
NW_1	11473544 (83.4%)	985245 (7.2%)	8112 (0.1%)	1282771 (9.3%)	2750 (0.0%)
NW_2	12408995 (82.4%)	1058145 (7.0%)	6636 (0.0%)	1573743 (10.5%)	3009 (0.0%)
NW_3	11782503 (88.8%)	662257 (5.0%)	6470 (0.0%)	812656 (6.1%)	2656 (0.0%)
NW_4	50742827 (87%)	2974477 (5.1%)	51391 (0.1%)	4493955 (7.7%)	46630 (0.1%)
NW_5	14085422 (84.7%)	1193487 (7.2%)	44280 (0.3%)	1267265 (7.6%)	29935 (0.2%)
OBF_1	13758992 (86.8%)	1003312 (6.3%)	47672 (0.3%)	998818 (6.3%)	39636 (0.3%)
OBF_2	11462047 (88.6%)	578220 (4.5%)	4453 (0.0%)	896470 (6.9%)	2591 (0.0%)
OBF_3	16205258 (85%)	1343711 (7.0%)	53216 (0.3%)	1430500 (7.5%)	30517 (0.2%)
OBF_4	15059598 (87%)	1027932 (5.9%)	8071 (0.0%)	1220164 (7.0%)	3466 (0.0%)
OBF_5	10889211 (82.2%)	738989 (5.6%)	7538 (0.1%)	1599515 (12.1%)	3969 (0.0%)

**Fig. 1:**
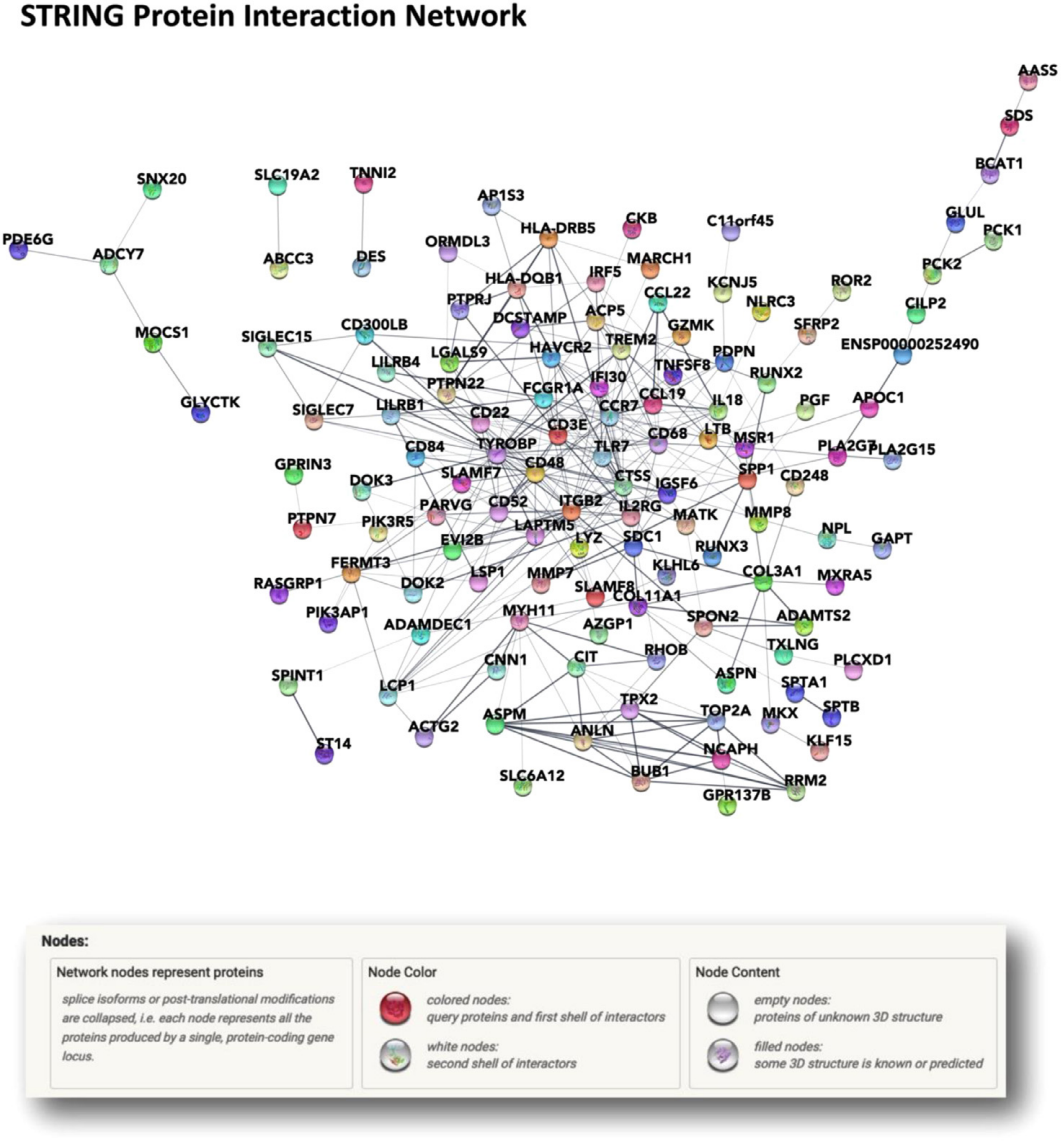
The STRING database was used to construct an interaction network of deregulated genes where the nodes are proteins, and the edges represent the predicted functional associations. The combined score is computed by combining the probabilities from the different evidence channels and corrected for the probability of randomly observing an interaction

Differential expression analysis performed with DESeq2 on R Studio returned 171 deregulated genes and among them, 52.63% had previously been associated to obesity (Supplementary Table 1). The STRING database is a publicly online database dedicated to protein association network that allowed the construction of an interaction network of the deregulated genes (Fig. 1).

**Fig. 2:**
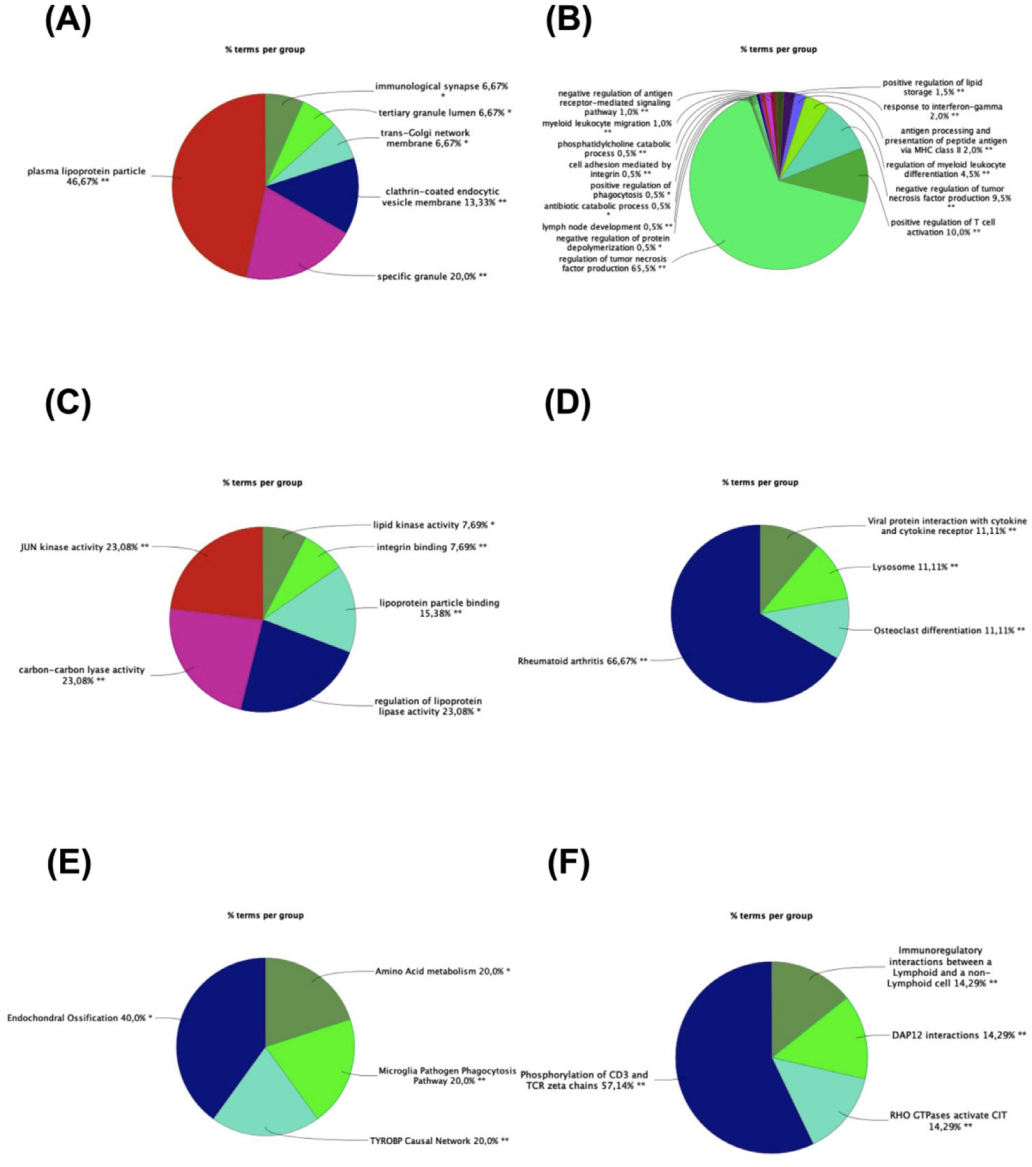
GO Cellular Component (A), GO Biological Process (B), GO Molecular Function (C), KEGG (D), Reactome (E), and WikiPathways (F) in obese vs. normal weight women. Each pie segment refers to the % of terms present per group (*p<0.05, **p<0.01 vs normal weight)

Pathways analysis performed on g:Profiler, Enrichr, ClueGO and GSEA allowed to gain mechanistic insights on gene expression generated by RNA-Seq analysis. gProfiler and Enrichr are online webtools that perform functional enrichment analysis on an input gene list that allowed to investigate the role of deregulated genes only [1], while GSEA was performed via clusterProfiler R package [2] to evaluate the perturbation due to whole gene expression alteration occurring in subcutaneous adipose tissue. Both analyses were executed considering gene ontologies, KEGG, Reactome and WikiPathways and deregulated pathways are reported in Fig. 2 and Supplementary Tables 2-4.

## 2 Experimental Design, Materials and Methods

### 2.1 Adult human adipose tissue collection, RNA extraction and quality assessment

Surgical biopsies of whole abdominal SAT tissues were collected from five obesity affected patients during bariatric surgery procedures and from five normal weight women. 500 mg of frozen subcutaneous adipose tissue was homogenized in RLT buffer (Qiagen), and RNA extracted using the RNeasy Mini Kit (Qiagen) according to the manufacturer's instructions (Qiagen). NanoDrop ND-1000 spectrophotometer (NanoDrop Technologies, USA) was used to determine both the concentration and RNA quality, whereas gel-electrophoresis was used to observe RNA degradation and impurity.

### 2.2 Libraries preparation and sequencing

RNA-seq stranded libraries were prepared with the CORALL Total RNA-Seq Library Prep Kit (Lexogen, Vienna, Austria) using 150 ng total RNA. The RiboCop rRNA Depletion Kit (Lexogen, Vienna, Austria) was used to remove rRNA. Qualities of sequencing libraries were assessed with D1000 ScreenTape Assay using the 4200 TapeStation System (Agilent, Santa Clara, CA, USA) and quantified with Qubit™ dsDNA HS Assay Kit (Invitrogen, Carlsbad, CA, USA). RNA processing was carried out using Illumina NextSeq 500 Sequencing. FastQ files were generated via llumina bcl2fastq2 (v. 2.17.1.14; https://support.illumina.com/downloads/bcl2fastq-conversion-software-v2-20.html) starting from raw sequencing reads produced by Illumina NextSeq sequencer.

### 2.3 Raw data processing and differential expression analysis

Genes and transcripts abundance was obtained using the BlueBee® Genomics Platform (Lexogen, Vienna, Austria). The CORALL Data Analysis pipeline on the BlueBee® Genomics Platform processes FASTQ generated by Illumina NextSeq sequencer through Unique Molecular Identifiers (UMI) extraction, trimming, alignment and quality control steps. As CORALL libraries contain N12 UMI at the start of Read 1, in the first step UMI are removed through UMI tools software. Then, adapter sequences, poly(A) sequences at the 3′ end of Read 1 and poly(T) sequences the 5′ end of Read 2 are trimmed through cutadapt software. After UMI extraction and trimming, trimmed reads are aligned through STAR aligner to the reference human genome. Differential expression analysis was performed using R package DESeq2 [3]. For each sample, the counts table was obtained from the “comp_frags_expt” column of the genes’ summary output file of BlueBee® Genomics Platform. The standard differential expression analyses steps are wrapped into a single function, DESeq. Tables with deregulated transcripts were obtained using the function “results”, which extracts a table with log_2_(condition sample/control sample) (e.g., log_2_FC), p values stat, and adjusted p values [3]. Genes were considered differentially expressed (DEGs) and retained for further analysis with |log_2_FC | ≥ 1 and a False Discovery Rate (FDR) ≤ 0.1.

### 2.4 Pathway analysis

Functional enrichment analysis was performed for Kyoto Encyclopedia of Genes and Genomes (KEGG) (https://www.genome.jp/kegg/) pathway analysis, WikiPathways analysis (https://www.wikipathways.org/index.php/WikiPathways) and Reactome (https://reactome.org/). Moreover, Gene Ontology (GO) analysis for biological processes, cellular components and molecular function [5] were executed. To perform enrichment analysis, two different approaches were used: over-representation analysis (ORA) and gene set enrichment analysis (GSEA). For ORA two web-tools on DEGs (g:Profiler [1] https://biit.cs.ut.ee/gprofiler/gost; Enrichr [4] https://maayanlab.cloud/Enrichr/) and ClueGO (a Cytoscape plug-in developed to facilitate the biological interpretation and to visualize functionally grouped terms [6], http://www.cytoscape.org/) with the DEGs being considered whereas GSEA was performed on R to evaluate perturbations due to all changes in gene expression and not only in DEGs. For Enrichr, the list of DEGs was inserted in the online webtool and the table with deregulated pathways ranked for significance for each database was downloaded and is reported in Supplementary Table 3. In the meantime, functional enrichment analysis was performed also through g:Profiler webtool using the hypergeometric test. As input gene list, the list of differentially expressed genes ranked for decreasing |log_2_FC| was used. All known genes were used as statistical domain scope and pathways were considered statistically significative if p-value according to Benjamin-Hochberg correction was lower than 0.05 (Supplementary Table 3). For ClueGO, the plug in was run and the list of DEGs was loaded as input. A medium network specificity showing only pathways with p.value lower than 0.05 was used (Fig. 2). GSEA was performed on clusterProfiler R package [2]. Given a priori defined set of gene S, GSEA determine whether the members of S are randomly distributed throughout the ranked gene list (L) or primarily found at the top or bottom [2]. The ranked gene list L was obtained as ranked list according to the “stat” column found in the table extracted from DESeq2 analysis. Gene set from Molecular Signature databases such as curated gene set (C2) and ontology gene sets (C5) were considered as priori set of gene S [2] and a p-value cut off of 0.05 for statistical significance. Pathways were up- or down-regulated according to the enrichment score (ES) which represents the degree to which a set S is over-represented at the top or bottom of the ranked list L. The score is calculated by looking at the list L, increasing a running-sum statistic when a gene in S is found and decreasing when it is not. The magnitude of the increment depends on the gene statistics such as the correlation of the gene with the phenotype (Supplementary Table 4).

### 2.5 Obesity-correlation identification

To identify the presence of genes previously related to obesity, for differentially expressed genes a bibliographic search of the gene name with the term “obesity” was performed and, when present, the pertaining article was considered as reference of the correlation (Supplementary Table 1).

### 2.6 STRING network construction

The STRING database was used to construct an interaction network of deregulated genes where the nodes are proteins, and the edges represent the predicted functional associations. The differentially expressed genes were loaded to the STRING database in the multiple proteins section, as a list of name, (https://string-db.org/cgi/input?sessionId=b1Lu1HpaOQpr&input_page_active_form=multiple_identifiers). Advanced settings were kept as standard conditions (Network type: full STRING network; Required score: medium confidence (0.400); FDR stringency: medium (5 percent). The combined score is computed by combining the probabilities from the different evidence channels and corrected for the probability of randomly observing an interaction.

## Ethics Statement

The present work is in accordance with the Declaration of Helsinki, and it was approved by the Ethical Committee of IRCCS Istituto Auxologico Italiano (Ethical Committee approval code #2020_10_20_04). A signed informed consent was obtained from each enrolled patient for tissue sampling.

## CRediT Author Statement

**Letizia Messa:** Conceptualization, Data curation, Formal analysis, Writing – original draft; **Federica Rey:** Conceptualization, Data curation, Formal analysis, Writing – original draft; **Cecilia Pandini** and **Bianca Barzaghini:** Formal analysis, Methodology; **Giancarlo Micheletto:** Methodology, Resources; **Manuela Teresa Raimondi:** Supervision, Writing - review & editing, Funding acquisition; **Simona Bertoli** and **Cristina Cereda:** Supervision, Writing - review & editing;**Gianvincenzo Zuccotti:** Resources, Supervision, Funding acquisition; **Raffaella Cancello:** Supervision, Writing - review & editing; **Stephana Carelli:** Conceptualization, Supervision, Writing - original draft and review.

## Declaration of Competing Interest

The authors declare that they have no known competing financial interests or personal relationships which have or could be perceived to have influenced the work reported in this article.

## Appendix Supplementary materials

Image, application 1

Image, application 2
