# Supplementary material for: RNA-seq dataset of subcutaneous adipose tissue: Transcriptional differences between obesity and healthy women
Source: Data Brief. 2021 Nov 27;39:107647. doi: 10.1016/j.dib.2021.107647 (PMC8640228; doi:10.1016/j.dib.2021.107647)
Supplement: Supplementary file 2 [file mmc2.docx]

**Supplementary Table 1: Obesity associated DE RNAs.** Out of the 171 Differentially Expressed RNAs (DE RNAs) identified as those with |log2FC| ≥ 1 and an FDR ≤ 0.1, 90 genes (52.63%) had previously been associated to obesity. The first two columns report the name of the genes never-before associated and the respective fold change, whereas in column D all the DE RNAs are reported, and in column E an annotation of whether they had been previously associated to obesity (YES/NO). This was identified through the bibliographic search of the gene name + the term “obesity” and, when present, the article link was reported in column F.

**Supplementary Table 2: Enrichr.** Functional enrichment analysis of differentially expressed genes resulting Enrichr webtool. The table reports the term, the overlap (e.g., ratio between the number of differentially expressed genes present in a specific pathway and the total number of genes in that pathway), the p-value, the adjusted p-value according to multiple hypothesis testing, and the name of the differentially expressed genes

**Supplementary Table 3: gProfiler.** Functional enrichment analysis of genes resulting from g:Profiler webtool. The table reports the source, the term name and term id, the adjusted p value, the term size and the intersection size

**Supplementary Table 4: GSEA.** Gene set enrichment analysis resulting from clusterProfiler R package. The table reports the ID, the description, the enrichment score, the NES, the p-value, adjusted p-value according to multiple testing correction and the leading edge to indicate the percentage of genes contributing to the enrichment score.
